# Supplementary material for: Fabrication of Multilayer Molds by Dry Film Photoresist
Source: Micromachines (Basel). 2022 Sep 23;13(10):1583. doi: 10.3390/mi13101583 (PMC9608710; doi:10.3390/mi13101583)
Supplement: Supplementary file 1 [file micromachines-13-01583-s001.zip › micromachines-1892146-supplementary.pdf]

Supplementary Materials

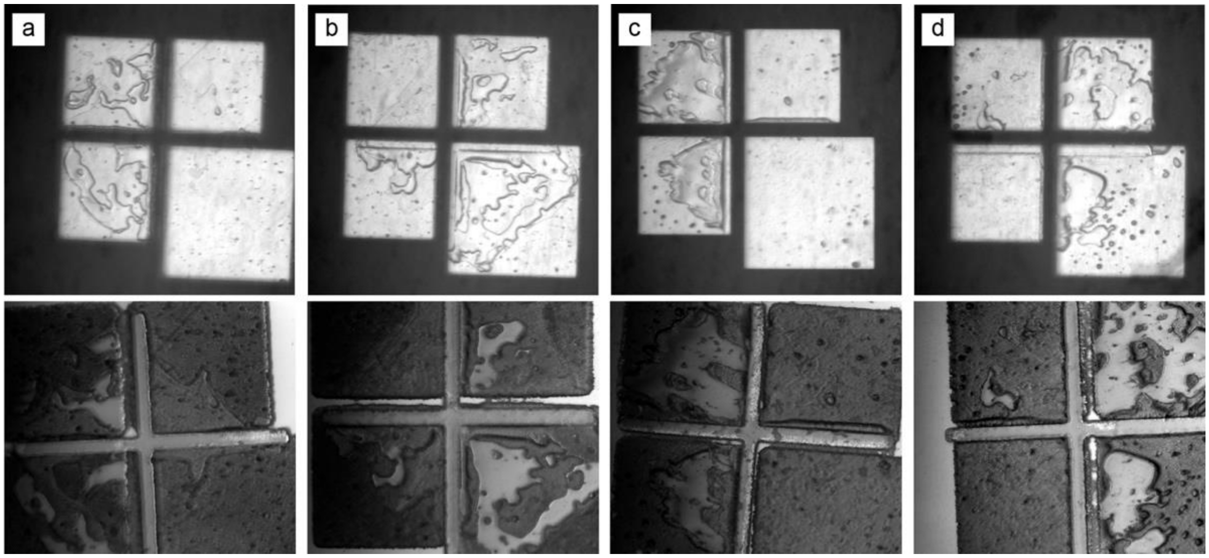

Figure S1. Full-wafer mask alignment. Microscope images of alignment marks during mask alignment (top row) and after development (bottom row). (a) Top-left corner. (b) Top-right corner. (c) Bottom-left corner. (d) Bottom-right corner. Here, the arms of the crosshairs are 33 μm, and thus a final alignment accuracy of better than 50 μm is achieved across the entire wafer.
